# Genetic Diversity and Biological Characteristics of H3 Avian Influenza Virus Isolated from China in 2021–2022 Showed the Emerging H3N8 Posed a Threat to Human Health

**DOI:** 10.1155/2024/9923259

**Published:** 2024-03-05

**Authors:** Xin Yin, Tiantian Wu, Shuo Liu, Cheng Peng, Jinping Li, Qiuyan Mao, Yaxin Zhang, Shuning Zhou, Wanting Zhou, Guangyu Hou, Wenming Jiang, Hualei Liu

**Affiliations:** China Animal Health and Epidemiology Center, Qingdao, China

## Abstract

The H3 influenza viruses are widespread in domestic poultry but have been ignored because their pathogenicity in poultry is low. Three human infections with H3N8 influenza viruses have been reported in China since 2022, raising public concern. Here, we comprehensively analyzed 30 H3 subtype avian influenza viruses isolated from live poultry markets in China between 2021 and 2022. Genetic and phylogenetic analyses showed that the H3 viruses have undergone frequent reassortment and have formed complex genotypes. Notably, the viruses that caused human infections in 2022–2023 were highly homologous to the H3N8 viruses circulating in poultry in 2022, with internal genes derived from the H9N2 viruses. The analysis of chicken infections indicated that the novel H3N8 viruses were more infectious in chickens than those that do not carry H9N2 genes, whereas the H3 viruses detected in China in 2021–2022 showed low pathogenicity in mice. Our findings suggest that the novel H3N8 viruses bearing internal H9N2 genes have adapted to and circulated in chickens and pose a threat to human health. These results highlight the need for continued surveillance of the H3 influenza viruses and their impact on the poultry industry.

## 1. Introduction

Avian influenza viruses (AIVs) pose a persistent threat to birds, mammals, and humans because of their rapid mutation, continuous reassortment, and cross-species transmission from birds to humans. They are divided into different subtypes based on the antigenicity of their two surface glycoproteins, hemagglutinin (HA) and neuraminidase (NA). Sixteen HA (H1–H16) and nine NA (N1–N9) subtypes have been detected in wild aquatic birds, and two novel HA subtypes (H17 and H18) and two novel NA subtypes (N10 and N11) have been identified in bats [1]. Only subtypes H5 and H7 have the potential to mutate from low-pathogenic forms to high-pathogenic forms that cause high mortality in domestic bird species. Over the past two decades, the highly pathogenic H5 viruses have not only caused severe outbreaks in domestic poultry and wild birds but have also caused more than 900 human infections in multiple countries [2, 3]. The H7N9 viruses were first reported to have infected and killed humans in China in 2013 [4]. Since then, they mutated to highly pathogenic forms in 2017 and caused outbreaks in chickens in China [5–9]. With the implementation of the H7N9 immunization policy for chickens, the number of human H7N9 infections in China has gradually decreased, with no new cases since 2020 [3, 10].

Low-pathogenic AIVs also pose a threat to human health. In recent years, H6N1, H3N8, H7N4, H9N2, H10N3, and H10N8 AIVs have also been shown to infect humans [11–15]. On April 26, 2022, a novel AIV infecting a 4-year-old boy in Henan Province, China, was identified as the first case of human H3N8 AIV infection. On May 19, 2022, a 5-year-old boy infected with H3N8 AIV was also reported in Hunan Province. On February 22, 2023, H3N8 AIV infection was reported in Guangdong Province in a 56-year-old woman who died on March 16. This was the first human death from H3N8 throughout the world. All three cases had a history of exposure to live poultry [14, 16, 17].

The H3 subtype influenza viruses have a wide range of hosts, including birds, poultry, swine, canines, equines, and humans [18–20]. H3 viral infections in poultry and wild birds rarely cause clinical symptoms, or the clinical symptoms are not obvious (often occurring as recessive infections). Several studies have reported the isolation of H3 subtype AIVs in wild birds, but the H3 subtype AIVs detected in poultry are usually from waterfowl and are rarely isolated from chickens [21, 22]. In this study, we analyzed several of the biological characteristics of 30 H3 subtype influenza viruses isolated during our routine surveillance of live poultry markets in 2021–2022. Our findings provide important information about the evolution and circulation of the H3 viruses in poultry in China and provide insights into the prevention and control of these viruses.

## 2. Materials and Methods

### 2.1. Virus Isolation and Identification

The H3 viruses used in this study were isolated from live poultry markets during routine surveillance between 2021 and 2022. All viruses were purified with three rounds of limiting dilution in 10-day-old specific-pathogen-free (SPF) embryonated chicken eggs and stored at −80°C.

### 2.2. Genetic and Phylogenetic Analyses

Viral RNA was extracted from the allantoic fluid with a FinePure Virus DNA/RNA Kit (Jifan, Suzhou, China). The HA gene was amplified with reverse transcription (RT)–PCR with specific primers, as previously described. The RT–PCR products were purified with an EZ Spin Column DNA Gel Extraction Kit (Sangon, Shanghai, China) and sequenced on an ABI 3730xl DNA Analyzer. The nucleotide sequences were edited with the SeqMan module of the DNAStar package. The whole gene sequence of the virus was blasted in the GISAID database, and the reference sequences that could reflect the genetic background of the virus were selected for phylogenetic analysis. A phylogenetic analysis of the eight segments' genes was performed with the ClustalW software and the MEGA 6.05 package using the neighbor-joining method. Bootstrap support was calculated with 1,000 replicates. A sequence identity cutoff of 95% was used to categorize the groups on the phylogenetic trees for each gene segment.

### 2.3. Studies in Chickens

Five-week-old SPF chickens (Vital River Laboratory Animal Technology Co., Ltd., Beijing, China) were used in this study. Groups of eight chickens were inoculated with 10^6.0^ median egg infective doses (EID_50_) per 0.1 ml of the test virus. All the chickens were monitored daily for clinical signs. Pharyngeal and cloacal swabs were taken from the live chickens at 2, 4, 6, and 8 days post-infection (dpi). At 4 dpi, three chickens from each group were killed, and the lungs, trachea, spleen, kidneys, brain, pancreas, and thymus were collected for viral titration in eggs. Sera were collected from chickens on 14 dpi for an HA inhibition antibody test.

### 2.4. Studies in Mice

Six-week-old female BALB/c mice (Beijing Vital River Laboratory Animal Technology Co., Ltd.) were used in this study. Groups of eight mice were lightly anesthetized with CO_2_ and inoculated intranasally with 10^6^ EID_50_ of each virus in a volume of 50 *μ*l. The weight and mortality of the mice were monitored for 14 consecutive days. To test the replication of the viruses in mice, groups of three mice were lightly anesthetized with CO_2_ and inoculated intranasally with 10^6^ EID_50_ of each virus in a volume of 50 *µ*l and then euthanized at 3 dpi. Their nasal turbinates, lungs, spleens, kidneys, and brains were collected and titrated for virus infectivity in eggs.

## 3. Results

### 3.1. Isolation and Genetic Analysis of H3 Viruses Collected in 2021–2022

**Figure 1:**
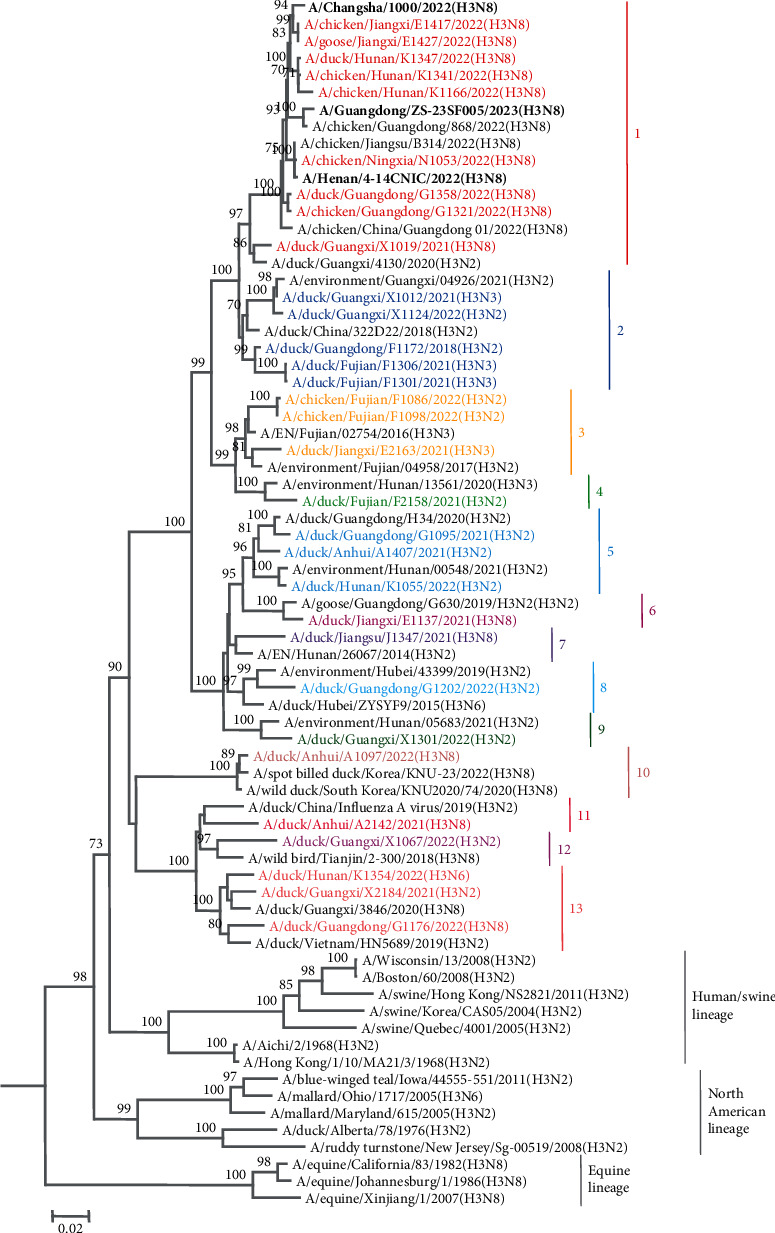
Phylogenetic tree of HA gene of H3 influenza viruses detected in 2021–2022. The phylogenetic tree of H3 was rooted with A/duck/Alberta/78/1976(H3N2). The H3N8 viruses that infect humans are shown in bold black. The trees were constructed with the MEGA 6.05 software using the neighbor-joining method. Bootstrap analysis was performed with 1,000 replicates.

The 30 H3 subtype AIVs used in this study were isolated from seemingly healthy geese, chickens, or ducks in live poultry markets during our routine surveillance in 2020–2021 and included 14 strains of H3N8 viruses, 11 strains of H3N2 viruses, one strain of H3N6 virus, and four strains of H3N3 virus (Appendix Table S1). The HA genes of the 30 H3 subtype AIVs were distinctly different from those of the human, swine, and equine H3 subtypes (Figure 1). A high degree of genetic diversity was observed among these H3 viruses, with nucleotide sequence identities ranging from 84.8% to 100%. A phylogenetic analysis of the HA gene revealed that these 30 H3 viruses could be further divided into 13 groups based on 95% nucleotide homology. The human infectious strains A/Changsha/1000/2022(H3N8), A/Henan/4-14CNIC/2022(H3N8) and A/Guangdong/ZS-23SF005/2023(H3N8) clustered in Group 1 and were highly homologous to the H3N8 viruses isolated in 2022, indicating that the H3N8 viruses that caused human infections in China in 2022 probably originated from the H3N8 viruses circulating in poultry.

**Figure 2:**
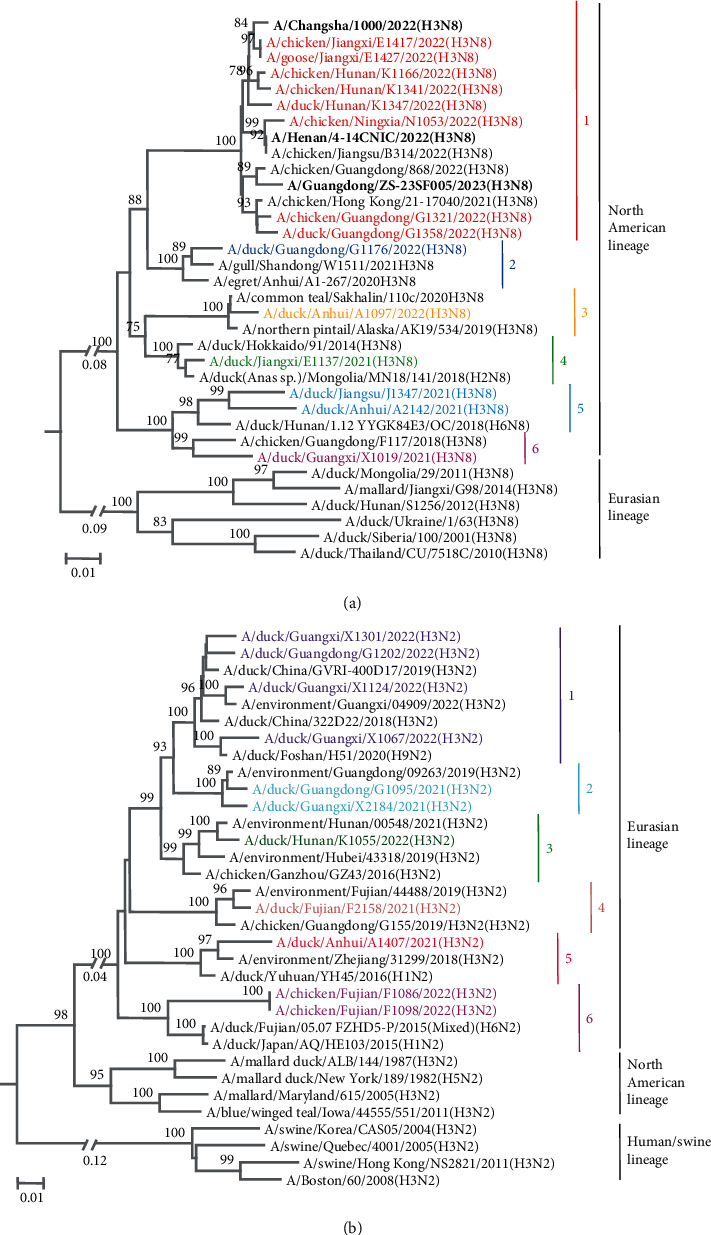
Phylogenetic trees of the NA gene of H3 influenza viruses detected in 2021–2022. The phylogenetic trees of N8 (a) and N2 (b) were rooted with A/equine/Johannesburg/1/1986(H3N8) and A/duck/Alberta/78/197(H3N2), respectively. The H3N8 viruses that infect humans are shown in bold black. The trees were constructed with the MEGA 6.05 software using the neighbor-joining method. Bootstrap analysis was performed with 1,000 replicates.

The N8 genes of the 14 strains of the H3N8 viruses shared 90.9%–99.7% identity at the nucleotide level and formed six groups based on 95% nucleotide homology. The N8 genes of human infectious strains A/Changsha/1000/2022(H3N8), A/Henan/4-14CNIC/2022(H3N8), and A/Guangdong/ZS-23SF005/2023(H3N8) were also highly homologous to the N8 genes of the H3N8 viruses isolated from chickens and ducks in 2022 (Figure 2(a)). The N2 genes of 11 strains of H3N2 viruses shared 89.6%–100% identity at the nucleotide level and formed six groups (Figure 2(b)). The N3 genes of four H3N3 strains shared 93.2%–99.8% identity at the nucleotide level, formed two groups, and were closely related to the NA genes of the H7N3 and H10N3 viruses isolated in China (Appendix Figure S1(g)). The NA gene of A/duck/Hunan/K1354/2022(H3N6) was highly homologous to wild bird strain A/Mallard(*Anas platyrhynchos*)/South Korea/KNU2021-46/2021(H4N6), with 99.43% homology (data not shown).

The six internal genes of the 30 H3 viruses showed distinct diversity, with the PB2, PB1, PA, NP, M, and NS genes sharing 84.4%–100%, 87.1%–100%, 87.3%–100%, 87.3%–100%, 89.6%–100%, and 86.4%–100%, respectively. They formed 7, 7, 10, 12, 3, and 5 groups on the phylogenetic trees, respectively (Appendix Figure S1(a)–S1(f)). Groups 1 and 2 in PB2, PB1, PA, and NP phylogenetic trees and Group 1 in M and NS phylogenetic trees all clustered with H9N2 viruses.

**Figure 3:**
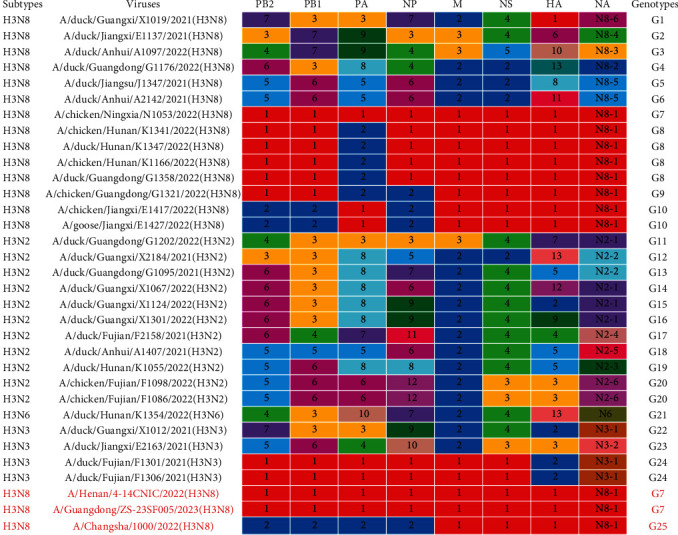
Genotypes of H3 viruses analyzed in this study.

Based on the combination of groups, we classified the 30 viruses into 24 distinct genotypes (Figure 3). The H3N8 viruses bearing H9N2 internal genes were classified into G7–G10 genotypes (Cui et al. [23] classified the H3N8 viruses bearing H9N2 viruses into 10 genotypes). A/Henan/4-14CNIC/2022(H3N8) and A/Guangdong/ZS-23SF005/2023(H3N8) can be classified as G7 genotypes (G1 in [23]), while A/Changsha/1000/2022(H3N8) (classified at G25 in Figure 3, G15 in [23]) is not among the 24 genotypes. Notably, the two H3N3 viruses classified as G24 shared the same internal gene combination as the G7 genotype.

Several key molecular markers were observed in each segment, which are associated with changes in viral receptor binding, replication, pathogenicity, and transmissibility. All 30 H3 viruses had the same amino acid motif, PEKQTR/GLF, at the cleavage site of the HA gene, indicating that all 30 H3 viruses were weakly pathogenic in chickens. Mutations A588V in PB2, I368V in PB1, K35R in PA, which increase the replication or virulence of AIVs in mammalian hosts, and S31N in M2, which increases resistance to amantadine and rimantadine, were observed in the eight H3N8 viruses isolated in 2022 and two H3N3 viruses isolated in 2021, and are attributed to recombination with the H9N2 subtype [24–27] (Table 1). Variants 155T in HA (H3 numbering), 622G in PB1, 383D in PA, 184K in NP, and 30D and 215A in M1 were conserved across all strains [28–32]. The substitution E627K in PB2 is the best-known mutation and is associated with increased viral fitness in mammals [30, 33]. However, in this study, no strain contained the E627K mutation; two H3N3 strains (DK/FJ/F1301/21(H3N3) and DK/FJ/F1306/21(H3N3)) had the E627V mutation, which has been shown to increase the replication or virulence of AIVs in mammals [34] (Table 1).

### 3.2. Virulence of H3 Viruses in Chickens

To investigate the replication and pathogenicity of the novel H3N8 viruses in chickens, we selected two novel H3N8 viruses bearing the H9N2 internal genes isolated from different hosts, one H3N8 virus without H9N2 internal genes, and one H3N2 virus to do this experiment. Eight 6-week-old SPF chickens per group were inoculated with one of those viruses (10^6^ EID_50_). Pharyngeal and cloacal swabs were taken from the live chickens at 2, 4, 6, and 8 dpi. At 4 dpi, three chickens in each group were killed, and their organs (lungs, trachea, spleen, kidneys, pancreas, and thymus) were collected for viral titration. None of the chickens died or showed any clinical symptoms during the 14-day observation period.

Strain DK/JX/E1137/2021(H3N8) was detected in the cloacal swabs of one chicken at 2, 4, and 6 dpi but not in the pharyngeal swabs (Table 2). One of the five chickens had seroconverted, with a titer of 1log2. Similarly, strain DK/GD/G1202/2022(H3N2) was detected in the cloacal swabs of one chicken at 2, 4, and 6 dpi and was detected in the pharyngeal swabs of one chicken at 2 dpi and four chickens at 4 dpi. Four of the five chickens had seroconverted, with titers of 1log2–3log2. Strains CK/NX/N1053/2022(H3N8) and GS/JX/E1427/2022(H3N8) were detected in the pharyngeal swabs of all chickens at 2 and 4 dpi and of about half the chickens at 6 dpi. Virus shedding was detected in the cloacal swabs of most chickens in the CK/NX/N1053/2022(H3N8) group at 4 dpi, and three of five chickens inoculated with CK/NX/N1053/2022(H3N8) shed the virus via cloacae at 6 dpi. GS/JX/E1427/2022(H3N8) was detected in the cloacal swabs of two chickens at 2 and 4 dpi. All chickens in the two groups had seroconverted, with titers of 3log2–5log2.

**Figure 4:**
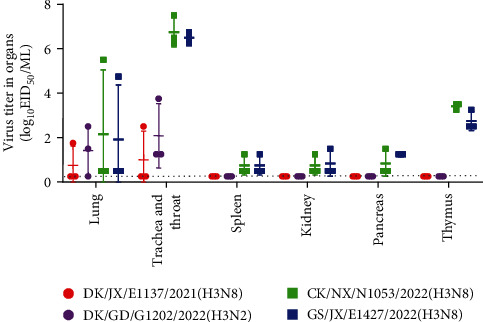
Replication of H3 viruses in chickens. Viral titers in the organs of chickens after inoculation with 10^6^ EID_50_ of different viruses. Three chickens from each group were euthanized at 3 dpi, and the viral titers in their organs were determined in eggs. Dashed lines indicate the lower limit of virus detection.

The replication of the four viruses in chickens varied. The DK/JX/E1137/2021(H3N8) virus was only detected in the lungs and throat of one chicken (Figure 4). The DK/GD/G1202/2022(H3N2) virus was detected in the lungs and throats of two chickens and three chickens, respectively. The CK/NX/N1053/2022(H3N8) and GS/JX/E1427/2022(H3N8) viruses that carried H9N2 internal genes replicated well in the throat and thymus, but the replication of the two strains was only detected in the lungs of one chicken. They were also detected in the spleen, kidneys, and pancreas of one or two chickens. These results indicate that the H3 viruses carrying internal H9N2 genes are more infectious in chickens than those that do not carry H9N2 genes.

### 3.3. Virulence of H3 Viruses in Mice

**Figure 5:**
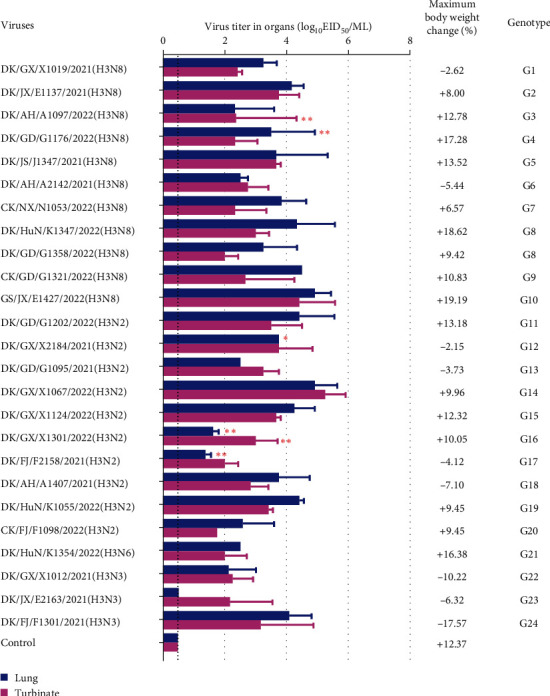
Replication and virulence of H3 viruses in mice. Viral titers in organs of mice after inoculation with 10^6^ EID_50_ of different viruses. The weight and mortality of the mice were monitored over a 14-day period. Three mice from each group were euthanized at 3 dpi, and viral titers in the organs were determined in eggs. Error bars represent standard deviations. The values labeled with one or two asterisks mean the virus was only detected in the organ of one or two mice, and the dashed lines indicate the lower limits of virus detection.

To understand the virulence of the H3 viruses, 25 H3 viruses, varying in the time of isolation, host, location, and genotype, were used to inoculate mice. A group of eight mice was inoculated intranasally with 10^6^ EID_50_ of each virus in a volume of 50 *µ*l. Three mice were killed at 3 dpi, and their nasal turbinates, lungs, spleens, kidneys, and brains were collected and titrated for viral infectivity in eggs. The remaining mice were weighed daily and observed for 14 days. All the mice survived during the 14-day observation period. The mice inoculated with DK/FJ/F1301/2021(H3N3) and DK/GX/X1012/2021(H3N3) lost 17.57% and 10.22% of their body weight, respectively (Figure 5). The mice inoculated with the other viruses showed only slight bodyweight loss (≤10%) or even gained weight (6.57%–19.19%). DK/JX/E2163/2021(H3N3) was only detected in the nasal turbinates, whereas the other viruses were all detected in the lungs and nasal turbinates. None of the strains was detected in the spleen, kidney, or brain.

## 4. Discussion

The H3 subtype is a low-pathogenic AIV subtype prevalent in domestic poultry. Here, we present a comprehensive analysis of 30 H3 subtype AIVs isolated from live poultry markets in China and have demonstrated that the H3 subtype viruses circulating in poultry have undergone frequent reassortment and formed complex genotypes. Notably, the novel H3N8 viruses circulating in poultry were highly homologous to the human H3N8 isolates, with their internal genes all derived from the H9N2 subtype virus.

Previous studies showed that the H3 influenza viruses did not form a stable lineage in poultry, with their gene segments originating from different subtypes of influenza viruses detected in wild birds or ducks [21]. H9 viruses are widely detected in live poultry markets and farms in China, resulting in the continuous recombination of H9 with other AIV subtypes [35, 36]. It is the optimal internal gene donor for emerging reassortant viruses that cause human infections, such as the H7N9, H10N3, and H10N8 viruses [4, 15, 37]. Therefore, we are concerned that the novel H3N8 viruses bearing H9N2 internal genes may become a stable branch and circulate in chickens or even cause sporadic human infections. Close and continuous surveillance of the H3 subtype of AIVs circulating in nature is required, and we must be aware of the impact of the H3 subtype on the poultry industry in the future.

Over the past 2 years, many agencies have reported the detection of novel H3N8 viruses in chicken farms and live poultry markets in China [14, 16, 17, 23, 38]. All the studies revealed that the novel H3N8 viruses formed independent lineages in the HA gene trees, with their internal genes being from poultry H9N2 viruses. Notably, Chen et al. [38] showed that the novel H3N8 viruses had been circulating in chickens for over a year at a high prevalence and had disseminated to at least seven provinces before detection in humans. We did not isolate the novel H3N8 viruses in 2021, but the two H3N3 viruses bearing the H9N2 internal genes similar to human strains were isolated in April 2021. The H3 subtype viruses, not just H3N8 subtypes, may have mixed with poultry H9N2 viruses very early on. Therefore, a retrospective analysis of H3 subtype strains isolated before 2021 needs to be conducted.

Previous studies have reported that the H3 subtype AIVs detected in poultry were predominantly from waterfowl, were rarely isolated from chickens, and rarely caused clinical symptoms [19, 21, 22]. Our study found that the novel H3N8 viruses bearing the H9N2 internal gene were more infectious in chickens. The virus replicates in more organs, such as the immune organ thymus, than just in the upper respiratory tract. Although challenged SPF chickens rarely show clinical symptoms under laboratory conditions, there have been cases of H3N8-related symptoms during the industrial production of chickens [39]. Recently, Mao et al. [40] reported the novel reassortant H3N3 AIVs bearing the HA gene of novel H3N8 viruses and internal genes of H9N2 showed increased pathogenicity in chicken, with viruses detected in the lungs, trachea, cecal tonsil, spleen, kidneys, pancreas, brain, bursa of fabricius, heart, liver, and thymus [40]. The internal genes derived from H9N2 viruses may play an important role in their infectivity in chickens, and the relevant mechanism requires further research.

The H1N1, H2N2, and H3N2 influenza viruses have caused influenza pandemics in humans, and H1N1 and H3N2 viruses are still circulating seasonally in humans. Guan et al. [21] reported that ferret antisera against human H3N2 viruses did not cross-react with any of the avian H3N2 viruses, suggesting that preexisting immunity does not limit the spread of the H3N2 avian viruses in humans. Sun et al. [16] and Zhu et al. [17] verified that the human population was immunologically naïve to the novel H3N8 viruses. The E627K mutation of PB2 is known to play a decisive role in the mammalian adaptation of AIVs [41, 42]. The A/Henan/4-10CNIC/2022(H3N8) virus contains the E627K mutation, whereas A/Changsha/1000/2022(H3N8) contains the E627V mutation, which has also been shown to increase the replication or virulence of AIVs in mammals [14, 34, 43]. The E627K mutation of PB2 was not observed in any of the H3 viruses in the present study; two strains that caused large weight loss in mice acquired the 627V mutation of PB2 in our study. Moreover, Cui et al. [23] reported the novel H3N8 isolated from poultry was transmissible between guinea pigs via respiratory droplets. Sun et al. [16] showed that the H3N8 virus isolated from humans had acquired the ability to transmit between respiratory droplets ferrets. Effective control measures must be established to reduce the prevalence of the virus in poultry, which also reduces the likelihood of infection in humans.

## Figures and Tables

**Table 1:** Molecular characteristics of the H3 viruses in this study.

Viruses	Mutations that increase the affinity to human-type receptor			Mutations that increase the replication or virulence of avian influenza viruses in mammalian hosts								Mutations that increase resistance to amantadine and rimantadine	
	HA			PB2			PB1	PA	NP	NS1		M2	
	I155T	Q226R	G228S	A588V	E627K	D701N	I368V	K356R	M105V	L103F	I106M	A30V	S31N
A/Henan/4-10CNIC/2022 (H3N8)	T	Q	S	V	K	D	V	R	V	L	I	A	N
A/Changsha/1000/2022(H3N8)	T	Q	G	V	V	D	V	R	V	L	I	A	N
A/Guangdong/ZS-23SF005/2023(H3N8)	T	Q	G	V	E	D	V	R	V	L	I	A	N
DK/GX/X1019/21(H3N8)	T	Q	G	A	E	D	I	K	V	F	M	A	S
DK/JX/E1137/21(H3N8)	T	Q	G	A	E	D	I	K	M	F	M	V	S
DK/AH/A1097/22(H3N8)	T	Q	G	A	E	D	I	K	M	L	M	A	S
DK/GD/G1176/22(H3N8)	T	Q	G	A	E	D	I	K	V	F	M	A	S
DK/JS/J1347/21(H3N8)	T	Q	G	A	E	D	I	K	M	F	M	A	S
DK/AH/A2142/21(H3N8)	T	Q	G	A	E	D	I	K	M	F	M	A	S
CK/NX/N1053/22(H3N8)	T	Q	G	V	E	D	V	R	V	L	I	A	N
CK/HuN/K1341/22(H3N8)	T	Q	G	V	E	D	V	R	V	L	I	A	N
DK/HuN/K1347/22(H3N8)	T	Q	G	V	E	D	V	E	V	L	I	A	N
CK/HN/K1166/22(H3N8)	T	Q	G	V	E	D	V	R	V	L	I	A	N
DK/GD/G1358/22(H3N8)	T	Q	G	V	E	D	V	R	V	L	I	A	N
CK/GD/G1321/22(H3N8)	T	Q	G	V	E	D	V	R	V	L	I	A	N
CK/JX/E1417/22(H3N8)	T	Q	G	V	V	D	V	R	V	L	I	A	N
GS/JX/E1427/22(H3N8)	T	Q	G	V	V	D	V	R	V	L	I	A	N
DK/GX/X2184/21(H3N2)	T	Q	G	A	E	D	I	K	V	F	M	A	S
DK/GD/G1202/22(H3N2)	T	Q	G	A	E	D	I	K	V	F	M	A	S
DK/GD/G1095/21(H3N2)	T	Q	G	A	E	D	I	K	V	F	M	A	S
DK/GX/X1067/22(H3N2)	T	Q	G	A	E	D	I	K	M	F	M	A	S
DK/GX/X1124/22(H3N2)	T	Q	G	A	E	D	I	K	M	F	M	A	S
DK/GX/X1301/22(H3N2)	T	Q	G	A	E	D	I	K	M	F	M	A	S
DK/FJ/F2158/21(H3N2)	T	Q	G	A	E	D	I	K	M	F	M	A	S
DK/AnH/A1407/21(H3N2)	T	Q	G	A	E	D	V	K	M	F	M	A	S
DK/HuN/K1055/22(H3N2)	T	Q	G	A	E	D	I	K	M	F	M	A	S
CK/FJ/F1098/22(H3N2)	T	Q	G	A	E	D	I	K	V	F	M	A	S
CK/FJ/F1086/22(H3N2)	T	Q	G	A	E	D	I	K	V	F	M	A	S
DK/HN/K1354/22(H3N6)	T	Q	G	A	E	D	I	K	M	F	M	A	S
DK/GX/X1012/21(H3N3)	T	Q	G	A	E	D	I	K	M	F	M	A	S
DK/JX/E2163/21(H3N3)	T	Q	G	A	E	D	I	K	M	F	M	A	S
DK/FJ/F1301/21(H3N3)	T	Q	G	V	E	D	V	R	V	L	I	A	N
DK/FJ/F1306/21(H3N3)	T	Q	G	V	E	D	V	R	V	L	I	A	N

**Table 2:** Virulence of H3 viruses in chickens.^a^

Viruses	Virus shedding (log_10_EID_50_) (positive/total)						Death/total	Seroconversion (HI antibody titers) (positive/total)
	2 dpi		4 dpi		6 dpi			
	Pharynx	Cloacae	Pharynx	Cloacae	Pharynx	Cloacae		
DK/JX/E1137/2021(H3N8)	<	2.75 (1/8)	<	5.5 (1/8)	<	1.25 (1/5)	0/5	2 (1/5)
DK/GD/G1202/2022(H3N2)	3.5 (1/8)	3.5 (1/8)	2.7 ± 0.90 (4/8)	5.75 (1/8)	<	1.25 (1/5)	0/5	2–8 (4/5)
CK/NX/N1053/2022(H3N8)	4.47 ± 0.86	1.25 (1/8)	4.22 ± 1.72	1.46 ± 0.17 (7/8)	1.75 ± 0.66 (3/5)	2.83 ± 2.32 (3/5)	0/5	8–32 (5/5)
GS/JX/E1427/2022(H3N8)	4.78 ± 0.76	1.38 ± 0.18 (2/8)	4.72 ± 0.34	1.38 ± 0.18 (2/8)	1.5 ± 0.35 (2/5)	<	0/5	16–32 (5/5)

^a^Groups of eight chickens were inoculated with 10^6.0^ EID_50_ per 0.1 ml of test virus. All chickens were monitored daily for clinical signs. Pharyngeal and cloacal swabs were taken from live chickens at 2, 4, 6, and 8 days postinfection (dpi). <, No virus was detected in this sample.

## Data Availability

Data are present in supplementary information files and also available upon request.

## Supplementary Materials

Supplementary Materials Appendix Table S1. Information of H3 viruses detected in 2021–2022. Appendix Figure S1. Phylogenetic trees of PB2(A), PB1(B), PA(C), NP(D), M(E), NS(F), and N3(G) genes of H3 viruses. The H3N8 viruses infection with human are shown in bold black.Trees were constructed with MEGA6.05 software using the neighbor-joining method. Bootstrap analysis was performed with 1,000.
