# Supplementary material for: Genetic Diversity and Biological Characteristics of H3 Avian Influenza Virus Isolated from China in 2021–2022 Showed the Emerging H3N8 Posed a Threat to Human Health
Source: Transbound Emerg Dis. 2024 Mar 5;2024:9923259. doi: 10.1155/2024/9923259 (PMC12016811; doi:10.1155/2024/9923259)
Supplement: Supplementary Materials — Appendix Table S1. Information of H3 viruses detected in 2021–2022. Appendix Figure S1. Phylogenetic trees of PB2(A), PB1(B), PA(C), NP(D), M(E), NS(F), and N3(G) genes of H3 viruses. The H3N8 viruses infection with human are shown in bold black.Trees were constructed with MEGA6.05 software using the neighbor-joining method. Bootstrap analysis was performed with 1,000. [file 9923259.f1.docx]

Supplementary files for

Genetic diversity and Biological Characteristics of H3 avian influenza virus isolated from China in 2021-2022 showed the emerging H3N8 posed a threat to human health

**Table S1** Information of H3 viruses detected in 2021-2022.

| **No.** | **Virus** | **Abbreviation** | **Sample information** | | | | | | |
| --- | --- | --- | --- | --- | --- | --- | --- | --- | --- |
|  |  |  | **Sample type** | **Collected date** | **Province** | **Location** | **Species** | **Isolate ID** | **Genotypes** |
| 1 | A/duck/Guangxi/X1019/2021(H3N8) | DK/GX/X1019/21(H3N8) | H3N8 | 22-Mar,2021 | Guangxi | Poultry market | Duck | EPI_ISL_18280410 | G1 |
| 2 | A/duck/Jiangxi/E1137/2021(H3N8) | DK/JX/E1137/21(H3N8) | H3N8 | 29-Mar,2021 | Jiangxi | Poultry market | Duck | EPI_ISL_18281078 | G2 |
| 3 | A/duck/Anhui/A1097/2022(H3N8) | DK/AH/A1097/22(H3N8) | H3N8 | 28-Apr,2022 | Anhui | Poultry market | Duck | EPI_ISL_18289716 | G3 |
| 4 | A/duck/Guangdong/G1176/2022(H3N8) | DK/GD/G1176/22(H3N8) | H3N8 | 3-Mar,2022 | Guangdong | Poultry market | Duck | EPI_ISL_18289717 | G4 |
| 5 | A/duck/Jiangsu/J1347/2021(H3N8) | DK/JS/J1347/21(H3N8) | H3N8 | 20-Apr,2021 | Jiangsu | Poultry market | Duck | [EPI_ISL_18289718](https://platform.epicov.org/epi3/start/EPI_ISL/18289718) | G5 |
| 6 | A/duck/Anhui/A2142/2021(H3N8) | DK/AH/A2142/21(H3N8) | H3N8 | 12-Oct,2021 | Anhui | Poultry market | Duck | [EPI_ISL_18289724](https://platform.epicov.org/epi3/start/EPI_ISL/18289724) | G6 |
| 7 | A/chicken/Ningxia/N1053/2022(H3N8) | CK/NX/N1053/22(H3N8) | H3N8 | 24-Apr,2022 | Ningxia | Poultry market | Chicken | EPI_ISL_18289725 | G7 |
| 8 | A/chicken/Hunan/K1341/2022(H3N8) | CK/HuN/K1341/22(H3N8) | H3N8 | 25-Apr,2022 | Hunan | Poultry market | Chicken | EPI_ISL_18289726 | G8 |
| 9 | A/duck/Hunan/K1347/2022(H3N8) | DK/HuN/K1347/22(H3N8) | H3N8 | 25-Apr,2022 | Hunan | Poultry market | Duck | EPI_ISL_18289727 | G8 |
| 10 | A/chicken/Hunan/K1166/2022(H3N8) | CK/HN/K1166/22(H3N8) | H3N8 | 25-Apr,2022 | Hunan | Poultry market | chicken | EPI_ISL_18289728 | G8 |
| 11 | A/duck/Guangdong/G1358/2022(H3N8) | DK/GD/G1358/22(H3N8) | H3N8 | 3-Mar,2022 | Guangdong | Poultry market | Duck | EPI_ISL_18289990 | G8 |
| 12 | A/chicken/Guangdong/G1321/2022(H3N8) | CK/GD/G1321/22(H3N8) | H3N8 | 2-Mar,2022 | Guangdong | Poultry market | Chicken | EPI_ISL_18290023 | G9 |
| 13 | A/chicken/Jiangxi/E1417/2022(H3N8) | CK/JX/E1417/22(H3N8) | H3N8 | 12-Apr,2022 | Jiangxi | Poultry market | Chicken | EPI_ISL_18290024 | G10 |
| 14 | A/goose/Jiangxi/E1427/2022(H3N8) | GS/JX/E1427/22(H3N8) | H3N8 | 12-Apr,2022 | Jiangxi | Poultry market | Goose | EPI_ISL_18290042 | G10 |
| 15 | A/duck/Guangdong/G1202/2022(H3N2) | DK/GD/G1202/22(H3N2) | H3N2 | 3-Mar,2022 | Guangdong | Poultry market | Duck | EPI_ISL_18596219 | G11 |
| 16 | A/duck/Guangxi/X2184/2021(H3N2) | DK/GX/X2184/21(H3N2) | H3N2 | 9-Nov,2021 | Guangdong | Poultry market | Duck | EPI_ISL_18290112 | G12 |

**Table S1.** Information of H3 viruses detected in 2021-2022 (Continued).

| **No.** | **Virus** | **Abbreviation** | **Sample information** | | | | | | |
| --- | --- | --- | --- | --- | --- | --- | --- | --- | --- |
|  |  |  | **Sample type** | **Collected date** | **Province** | **Location** | **Species** | **Isolate ID** | **Genotypes** |
| 17 | A/duck/Guangdong/G1095/2021(H3N2) | DK/GD/G1095/21(H3N2) | H3N2 | 9-Mar,2021 | Guangdong | Poultry market | Duck | EPI_ISL_18290123 | G13 |
| 18 | A/duck/Guangxi/X1067/2022(H3N2) | DK/GX/X1067/22(H3N2) | H3N2 | 15-Mar,2022 | Guangxi | Poultry market | Duck | EPI_ISL_18290130 | G14 |
| 19 | A/duck/Guangxi/X1124/2022(H3N2) | DK/GX/X1124/22(H3N2) | H3N2 | 15-Mar,2022 | Guangxi | Poultry market | Duck | EPI_ISL_18290219 | G15 |
| 20 | A/duck/Guangxi/X1301/2022(H3N2) | DK/GX/X1301/22(H3N2) | H3N2 | 17-Mar,2022 | Guangxi | Poultry market | Duck | EPI_ISL_18291783 | G16 |
| 21 | A/duck/Fujian/F2158/2021(H3N2) | DK/FJ/F2158/21(H3N2) | H3N2 | 9-Nov,2021 | Fujian | Poultry market | Duck | EPI_ISL_18291807 | G17 |
| 22 | A/duck/Anhui/A1407/2021(H3N2) | DK/AnH/A1407/21(H3N2) | H3N2 | 13-Apr,2021 | Anhui | Poultry market | Duck | EPI_ISL_18292895 | G18 |
| 23 | A/duck/Hunan/K1055/2022(H3N2) | DK/HuN/K1055/22(H3N2) | H3N2 | 24-Apr,2021 | Hunan | Poultry market | Duck | EPI_ISL_18292972 | G19 |
| 24 | A/chicken/Fujian/F1098/2022(H3N2) | CK/FJ/F1098/22(H3N2) | H3N2 | 23-Mar,2022 | Fujian | Poultry market | Chicken | EPI_ISL_18292972 | G20 |
| 25 | A/chicken/Fujian/F1086/2022(H3N2) | CK/FJ/F1086/22(H3N2) | H3N2 | 23-Mar,2022 | Fujian | Poultry market | Chicken | EPI_ISL_18596681 | G20 |
| 26 | A/duck/Hunan/K1354/2022(H3N6) | DK/HuN/K1354/22(H3N6) | H3N6 | 25-Apr,2022 | Hunan | Poultry market | Duck | EPI_ISL_18294232 | G21 |
| 27 | A/duck/Guangxi/X1012/2021(H3N3) | DK/GX/X1012/21(H3N3) | H3N3 | 22-Mar,2021 | Guangxi | Poultry market | Duck | EPI_ISL_18294233 | G22 |
| 28 | A/duck/Jiangxi/E2163/2021(H3N3) | DK/JX/E2163/21(H3N3) | H3N3 | 14-Oct,2021 | Jiangxi | Poultry market | Duck | EPI_ISL_18294237 | G23 |
| 29 | A/duck/Fujian/F1301/2021(H3N3) | DK/FJ/F1301/21(H3N3) | H3N3 | 8-Apr,2021 | Fujian | Poultry market | Duck | [EPI_ISL_18294245](https://platform.epicov.org/epi3/start/EPI_ISL/18294245) | G24 |
| 30 | A/duck/Fujian/F1306/2021(H3N3) | DK/FJ/F1306/21(H3N3) | H3N3 | 8-Apr,2021 | Fujian | Poultry market | Duck | [EPI_ISL_18294484](https://platform.epicov.org/epi3/start/EPI_ISL/18294484) | G24 |

Figure S1. Phylogenetic trees of PB2(A), PB1(B), PA(C), NP(D), M(E), NS(F) and N3(G) genes of H3 viruses. The H3N8 viruses infection with human are shown in bold black.Trees were constructed with MEGA6.05 software using the neighbor-joining method. Bootstrap analysis was performed with 1000.

**
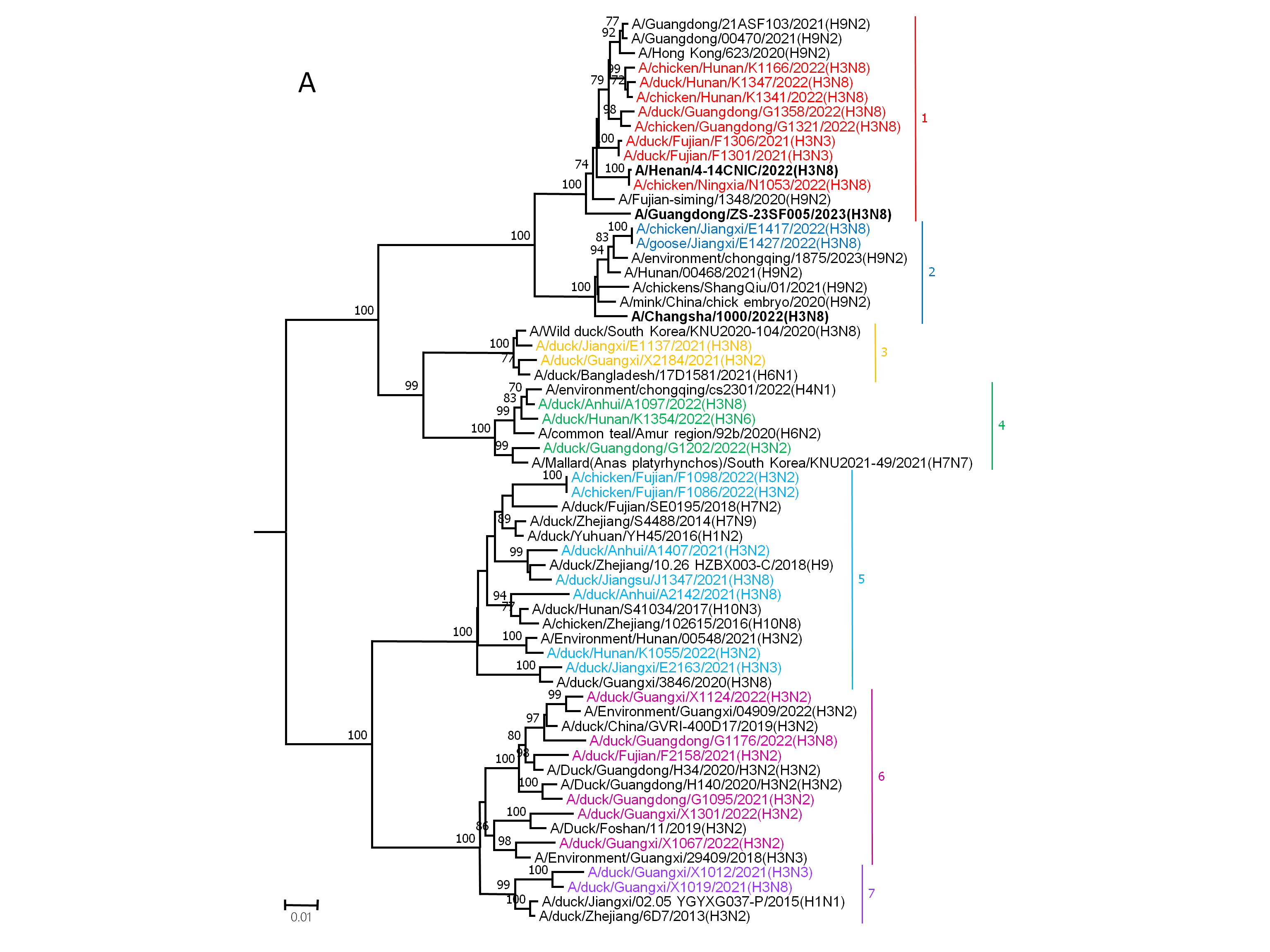

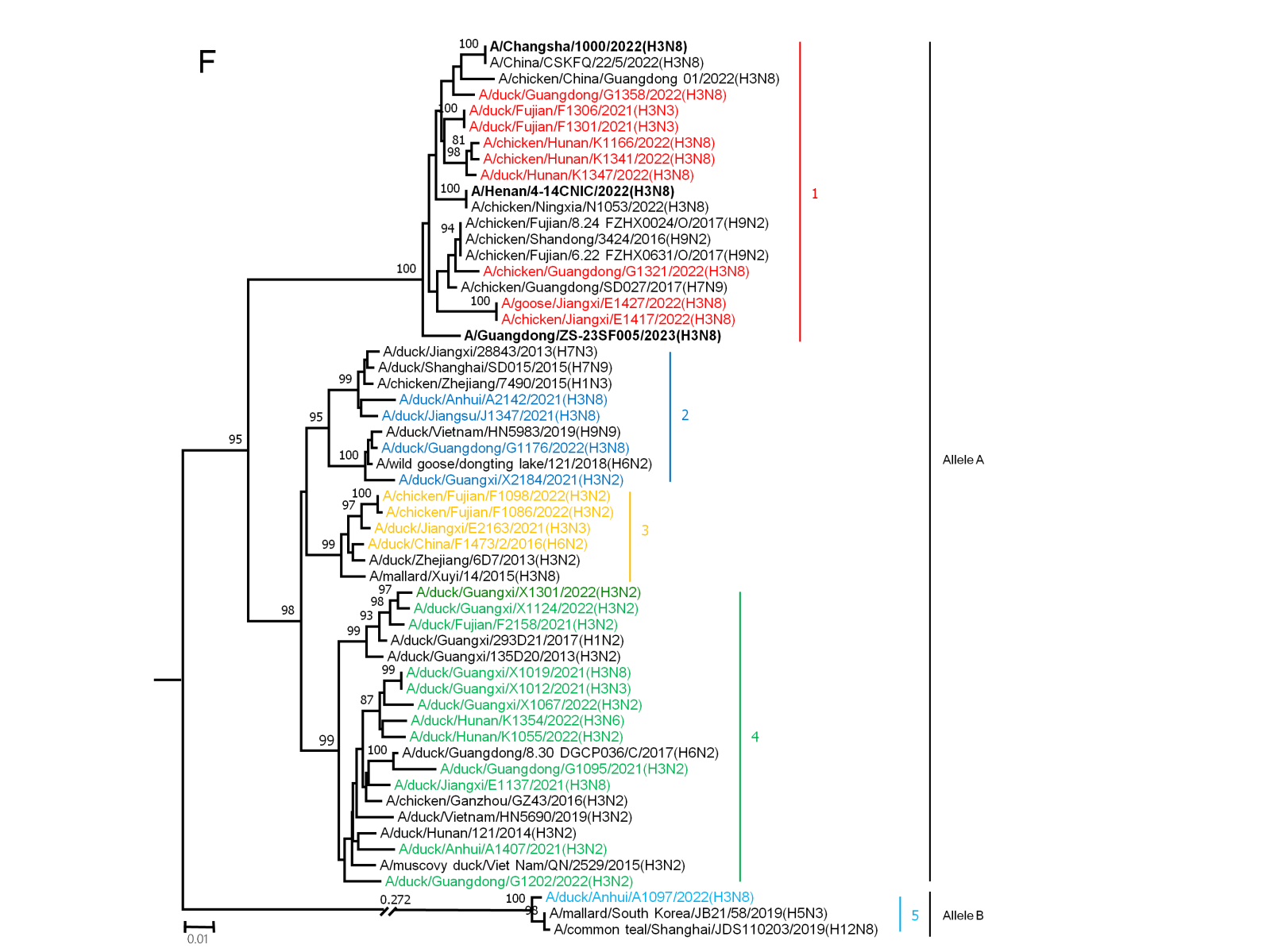
**

**
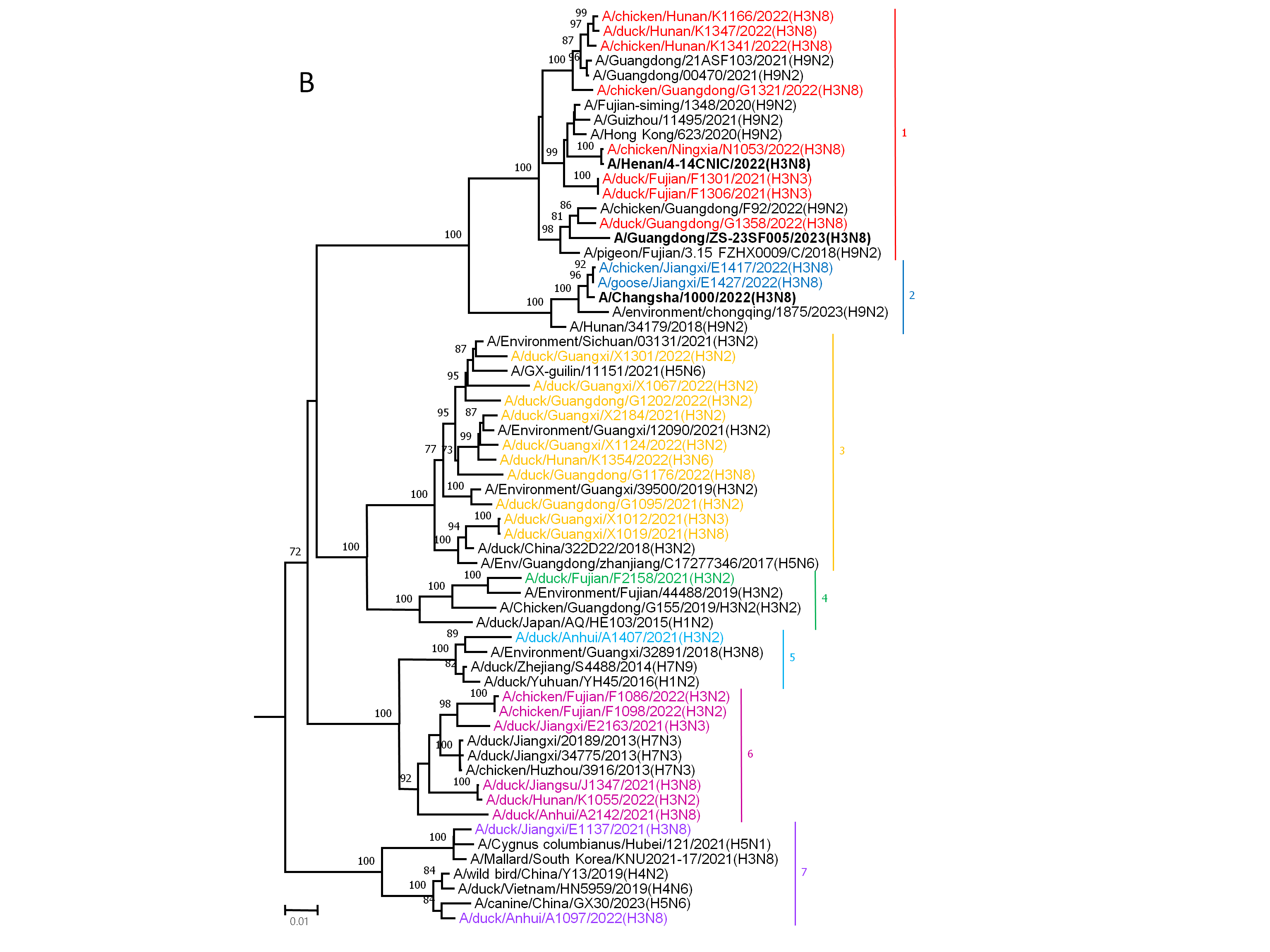
**

**
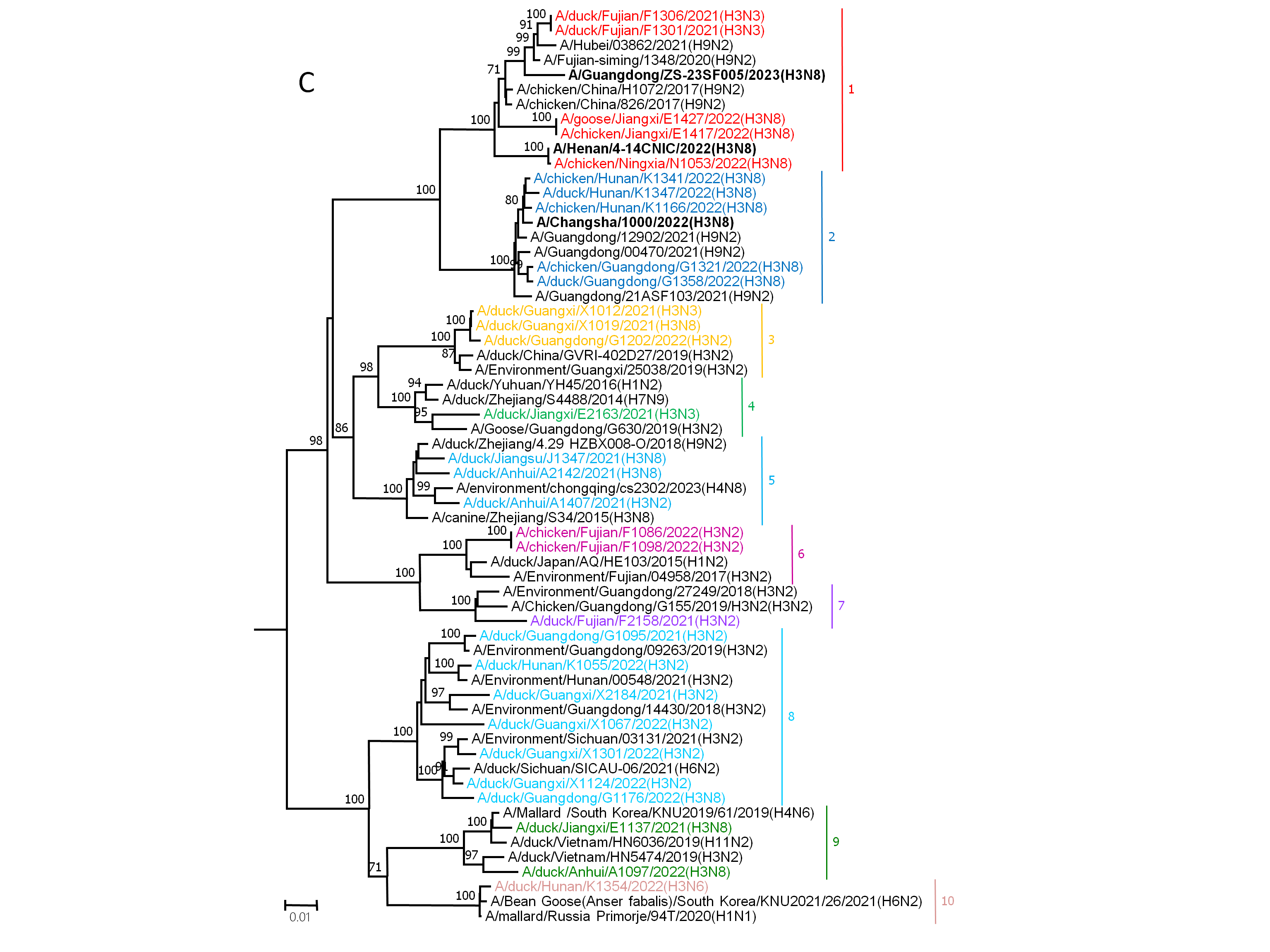
**

**
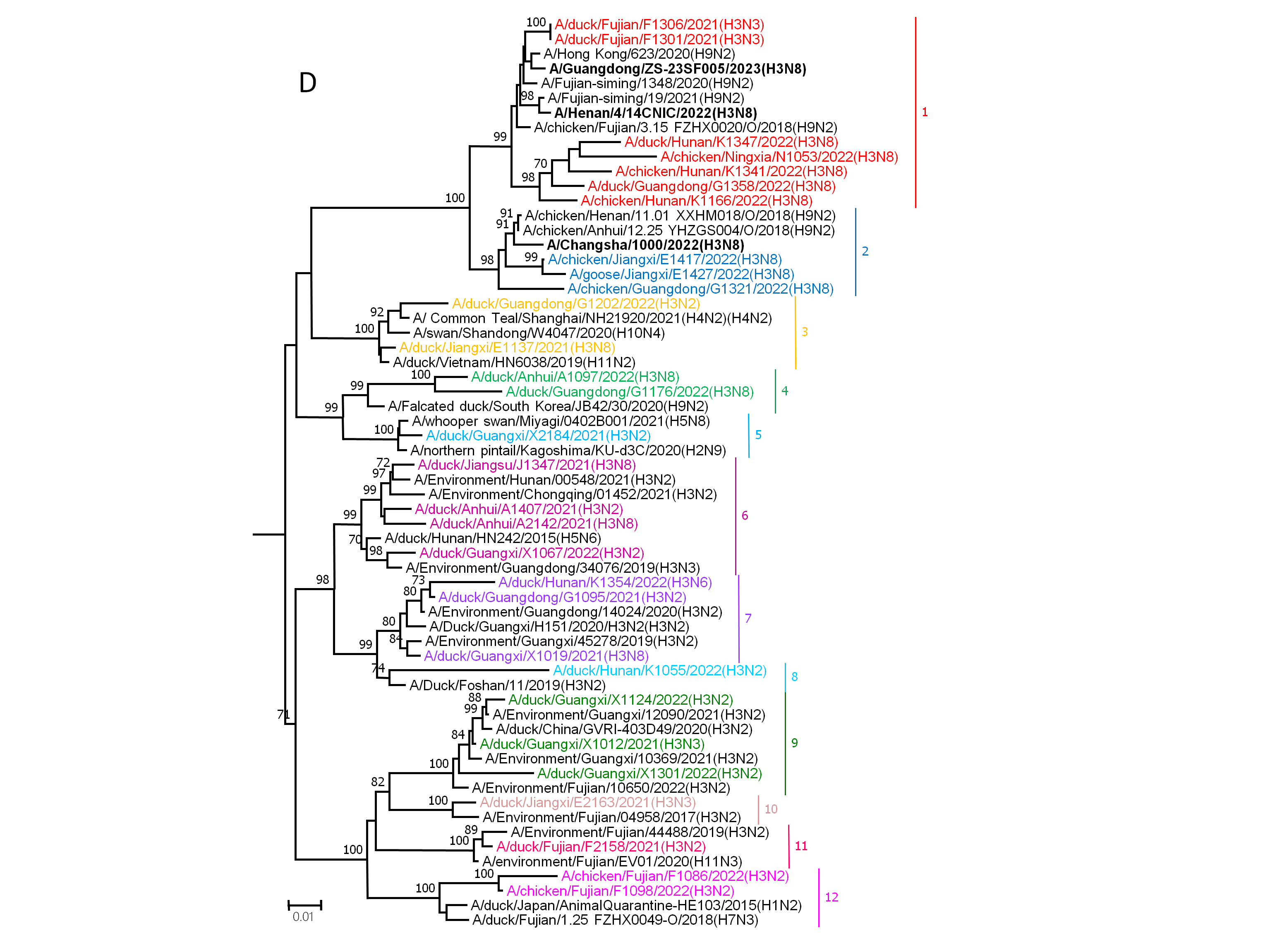
**

**
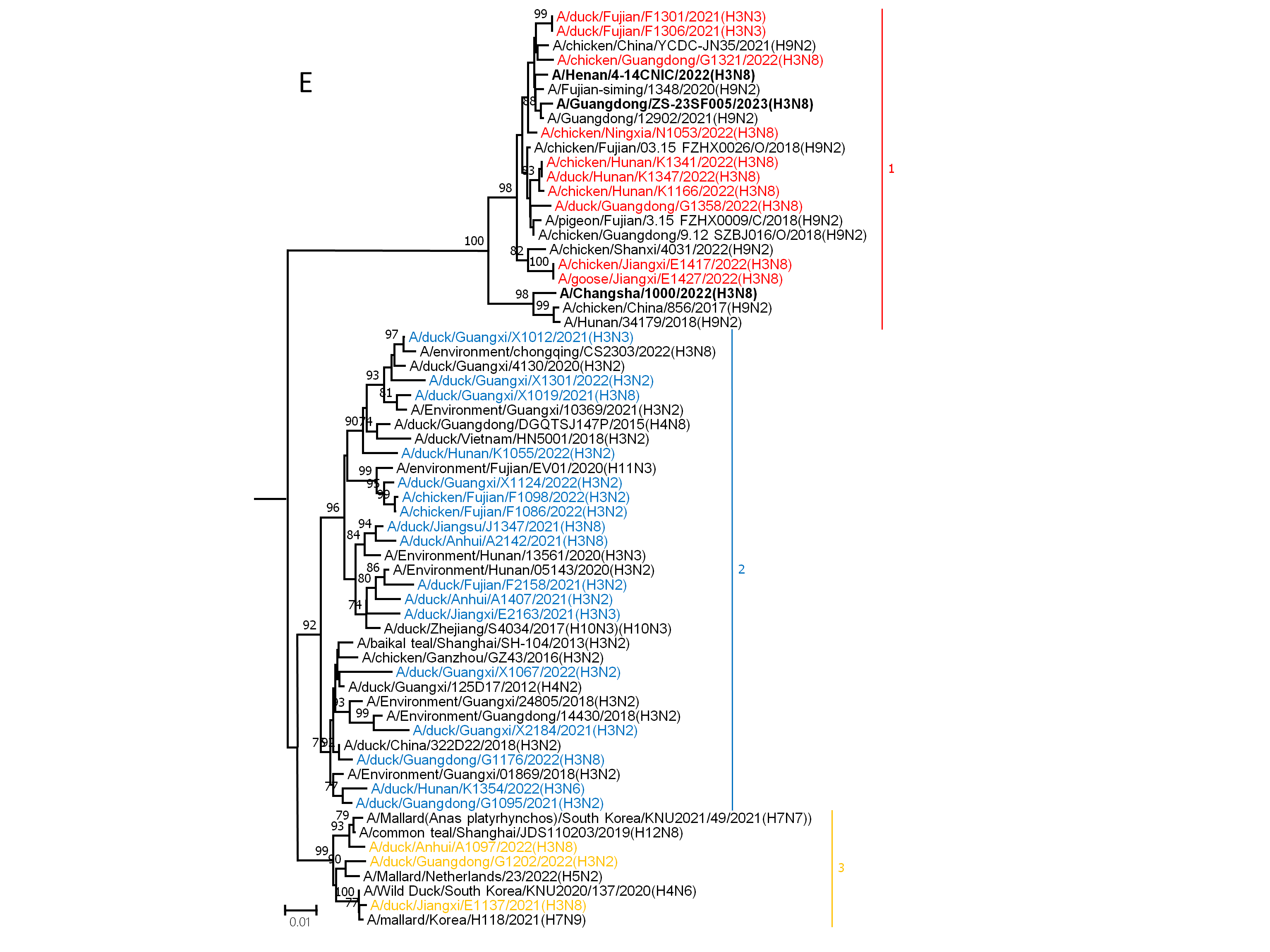
**

**
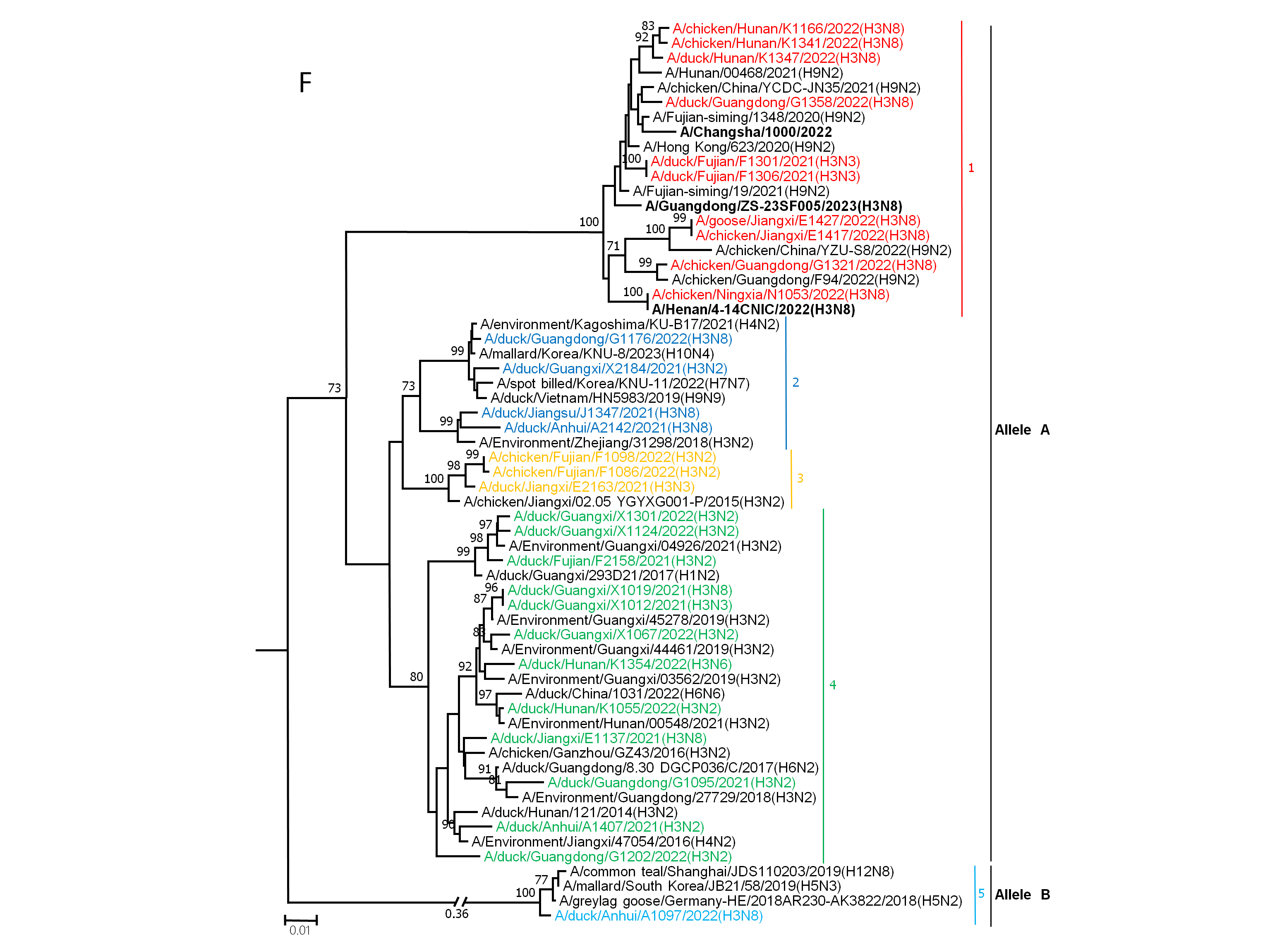
**

**
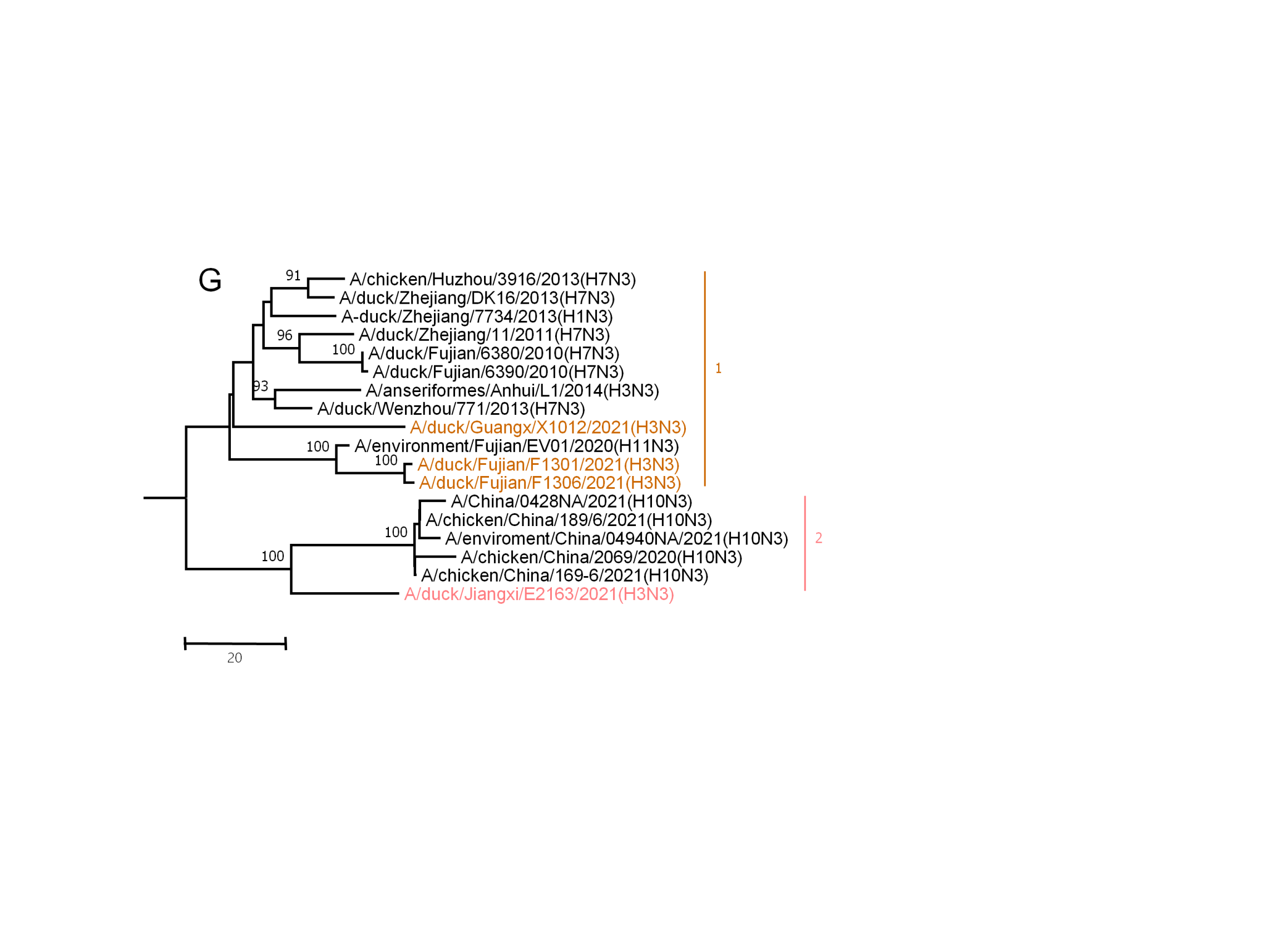
**
